# Survival of COVID-19 Patients With Respiratory Failure is Related to Temporal Changes in Gas Exchange and Mechanical Ventilation

**DOI:** 10.1177/08850666211033836

**Published:** 2021-08-16

**Authors:** Victoria J. Ende, Gurinder Singh, Ioannis Babatsikos, Wei Hou, Haifang Li, Henry C. Thode, Adam J. Singer, Tim Q. Duong, Paul S. Richman

**Affiliations:** 1 12300 Renaissance School of Medicine at Stony Brook University, Stony Brook, NY, USA; 2 205134 Jack D Weiler Hospital of the Albert Einstein College of Medicine Emergency Room, Bronx, NY, USA

**Keywords:** COVID-19, mechanical ventilation, oxygenation index, ventilatory ratio, Murray lung injury score, ICU outcomes

## Abstract

**Background:** Respiratory failure due to coronavirus disease of 2019 (COVID-19) often presents with worsening gas exchange over a period of days. Once patients require mechanical ventilation (MV), the temporal change in gas exchange and its relation to clinical outcome is poorly described. We investigated whether gas exchange over the first 5 days of MV is associated with mortality and ventilator-free days at 28 days in COVID-19. **Methods:** In a cohort of 294 COVID-19 patients, we used data during the first 5 days of MV to calculate 4 daily respiratory scores: PaO_2_/FiO_2_ (P/F), oxygenation index (OI), ventilatory ratio (VR), and Murray lung injury score. The association between these scores at early (days 1-3) and late (days 4-5) time points with mortality was evaluated using logistic regression, adjusted for demographics. Correlation with ventilator-free days was assessed (Spearman rank-order coefficients). **Results:** Overall mortality was 47.6%. Nonsurvivors were older (*P* < .0001), more male (*P* = .029), with more preexisting cardiopulmonary disease compared to survivors. Mean PaO_2_ and PaCO_2_ were similar during this timeframe. However, by days 4 to 5 values for all airway pressures and FiO_2_ had diverged, trending lower in survivors and higher in nonsurvivors. The most substantial between-group difference was the temporal change in OI, improving 15% in survivors and worsening 11% in nonsurvivors (*P* < .05). The adjusted mortality OR was significant for age (1.819, *P* = .001), OI at days 4 to 5 (2.26, *P* = .002), and OI percent change (1.90, *P* = .02). The number of ventilator-free days correlated significantly with late VR (−0.166, *P* < .05), early and late OI (−0.216, *P* < .01; −0.278, *P* < .01, respectively) and early and late P/F (0.158, *P* < .05; 0.283, *P* < .01, respectively). **Conclusion:** Nonsurvivors of COVID-19 needed increasing intensity of MV to sustain gas exchange over the first 5 days, unlike survivors. Temporal change OI, reflecting both PaO_2_ and the intensity of MV, is a potential marker of outcome in respiratory failure due to COVID-19.

## Introduction

Severe acute respiratory syndrome coronavirus-2 (SARS-CoV-2) has caused more than 79 million infections worldwide at the time of this writing, and over 1.7 million deaths.^1^ Approximately 10% to 20% of patients hospitalized with coronavirus disease 2019 (COVID-19) are admitted to intensive care unit (ICU) with severe hypoxemia and diffuse lung infiltrates; many progress to require mechanical ventilation (MV) for the acute respiratory distress syndrome (ARDS).^2-4^ The sheer volume of such cases threatens to fill the capacity of ICUs in many regions, even in resource-rich nations. Addressing this challenge will require a better understanding of the factors that predict poor clinical outcomes in mechanically ventilated COVID-19 patients.

The severity of hypoxemia, expressed as the PaO_2_/FiO_2_ (P/F) ratio is widely used to stratify ARDS into mild, moderate, and severe categories according to the Berlin definition.^5^ There is conflicting evidence regarding whether these categories reliably predict ARDS mortality.^6-8^ ARDS due to COVID-19 appears to have atypical features compared to other causes of ARDS. In COVID-19 disease, it is commonly observed that the severity of hypoxemia progresses to ARDS in an unusually slow fashion over several days. Some authors have observed an atypical ARDS presentation in a subset of COVID-19 patients with relatively preserved lung mechanics, while others have reported surprisingly little dyspnea in association with marked hypoxemia.^9,10^ The postulated mechanisms behind such atypical features are subjects of dispute. In view of this, it is unclear whether temporal trends in gas exchange in COVID-19 are predictive of outcome.

The goal of this study was to investigate whether temporal trends in gas exchange parameters over the initial days of MV are associated with mortality and ventilator-free days (VFDs) in COVID-19 patients with respiratory failure. We specifically tested the association of these clinical outcomes with the temporal changes in PaO_2_ /FiO_2_%/ratio (P/F), oxygenation index (OI), ventilatory ratio (VR), and the Murray lung injury score (MLIS).

## Methods

### Study Population

**Figure 1.**
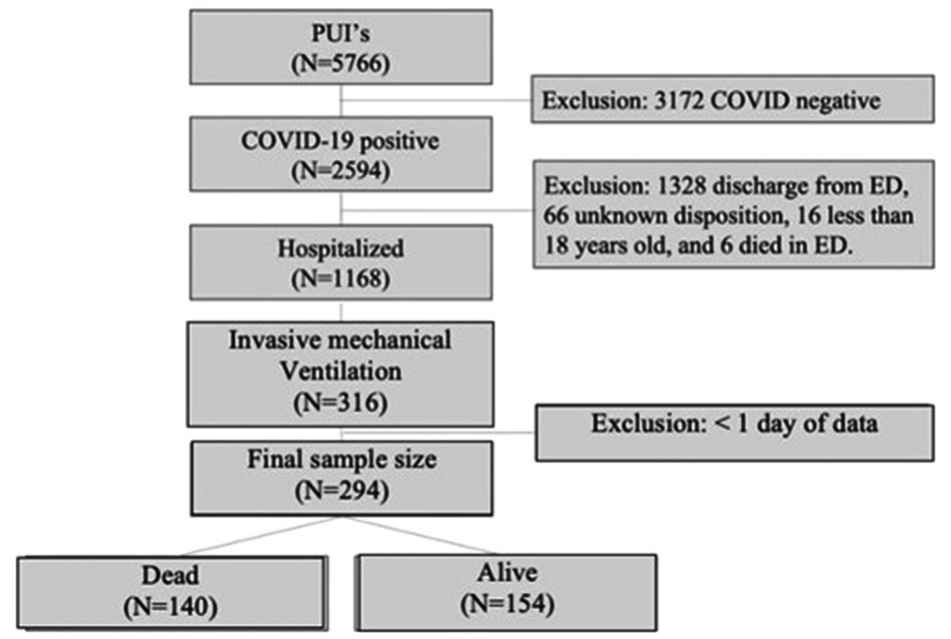
Flowchart of patient population included in this study. Two hundred ninety four patients were included in the final sample. The terms alive and dead refer to the patient’s status at time data collection was finalized, 60 days after all 294 patients had entered the study period.

This retrospective study was approved by the Institutional Review Board with an exemption for informed consent. Our study followed the Strengthening of Reporting of Observational Studies in Epidemiology (STROBE) reporting guidelines from cross-sectional studies.^11^ Data came from Stony Brook University Hospital Emergency Department (ED) COVID-19 Persons Under Investigation Registry from February 7, 2020, to June 30, 2020. The study population included all adults (>18 years) treated with MV in the hospital ICUs with a confirmed positive real-time polymerase chain reaction test for SARS-CoV-2 on a nasopharyngeal swab specimen. Patients were excluded if they expired in the ED, were still hospitalized at the time of data analysis over 2 months after ED admission for this cohort, or had received MV for <1 day. The study sample size was 294 patients (Figure 1).

### Outcome Measures

The study outcomes were in-hospital mortality and VFDs in the first 28 days after intubation. Patients were considered survivors if they were discharged alive from the hospital at the time of data analysis. Any patient who died within the 28 days or remained on MV for over 28 days received a 0 for VFD.

### Independent Variables

Demographics, major comorbidities, vital signs, and laboratory values were tabulated at ED admission. The earliest arterial blood gas (ABG) values on each of the first 5 days of MV and the closest corresponding ventilator parameters were recorded, including the fraction of inspired oxygen (FiO_2_%), mean airway pressure (MAP), positive end-expiratory pressure (PEEP), respiratory rate (RR), tidal volume (TV), and dynamic peak inspiratory pressure (PIP). ABG variables included pH, partial pressure of oxygen (PaO_2_), and partial pressure of carbon dioxide (PaCO_2_). After 5 days of MV, ABG data is obtained only intermittently at our institution, limiting our ability to analyze gas exchange for a more prolonged period.

We calculated the multivariable MLIS with a modified radiographic scoring method, used in a prior study of these registry patients,^12^ as follows: 3 thoracic radiologists scored the geographical extent of parenchymal lung infiltrates or consolidations in each lung separately on a 0-4 scale (0 = no involvement, 1 = <25%, 2 = 26%-50%, 3 = 51%-75%, 4 = >75% involvement). For each patient, the mean scores for the right and left lung were added together, divided by 2, and rounded to the nearest integer.^13^ Kappa scores for this method revealed an acceptable degree of interobserver agreement. It differs only slightly from the 4-quadrant system used in the classical Murray score.^13^

Calculated gas exchange scores were as follows^14,15^:

- VR = RR*TV*PaCO_2_/predicted body weight*100*37.5.
- OI = FiO_2_*MAP/PaO_2_.

The mean value of each score (MLIS, VR, OI, and P/F ratio) during days 1 to 3 and days 4 to 5 of MV were calculated separately, and referred to as early and late time points, respectively, as described in Results section.

### Statistical Analysis

Statistical analysis was performed using SPSS v26 (IBM, Armonk) and SAS v9.4 (SAS Institute, Cary). Group comparisons of categorical variables in frequencies and percentages were performed using the χ^2^ test or Fisher exact test. Group comparison of continuous variables in medians and interquartile ranges (IQR) used the Mann–Whitney *U*-test. Ventilation parameters were compared between survivors and nonsurvivors for the first 5 days on MV using repeated analysis of variance. The temporal differences in clinical variables between survivor and nonsurvivor groups were compared using a *t*-test. The associations between clinical variables and mortality were evaluated using logistic regression with odds ratios (ORs). We tested for collinearity between variables using the variance inflation factor. Standardized ORs were estimated in order to compare the associations of clinical variables with different units. Logistic regressions were adjusted for age, gender, and the presence of 2 comorbidities, hypertension (HTN) and coronary artery disease (CAD). Finally, Spearman rank-order correlations were estimated between VFDs and clinical variables. For all analyses, a *P* value of <.05 was considered to be statistically significant.

## Results

### Mortality Outcomes

The final sample size consisted of 294 COVID-19 positive patients receiving MV. Mortality was 47.6%. Table 1 shows the demographics, comorbidity, vital signs, ABG, and other laboratory values at admission. Patients in the nonsurvivor group were older (*P* < .0001), more male (*P* = .029), and more frequently had a history of CAD (*P* = .004), chronic obstructive pulmonary disease (COPD) (*P* = .042), and congestive heart failure (CHF) (*P* = .033) compared to the survivor group. They also had slightly lower mean values for body temperature and hemoglobin-O_2_ saturation, and a slightly higher mean respiratory rate. Notable differences in laboratory values included higher serum levels of B-type natriuretic peptide, creatinine, and D-dimer among nonsurvivors, as shown in Table 1.

**Table 1.** Demographics, Comorbidities, Vital Signs, and Laboratory Values of COVID-19 Survivors and Nonsurvivors on MV.

	Patients, no. (%)		
	Survivors (*n* = 154)	Nonsurvivors (*n* = 140)	*P* value
**Demographics**			
Age, median	57.5 (50.0, 67.0)	68.0 (57.5, 77.0)	<.0001***
**Gender**			.029*
Female	57 (37%)	35 (25%)	
Male	97 (63%)	105 (75%)	
**Race**			.58
White	78 (51%)	67 (48%)	
African American	11 (7%)	8 (6%)	
Other	10 (6%)	8 (6%)	
Unknown	55 (36%)	55 (39%)	
**Ethnicity**			.768
Hispanic	42 (27%)	33 (24%)	
Not Hispanic	90 (58%)	85 (61%)	
Unknown	22 (14%)	21 (15%)	
**Comorbidities**			
Hypertension	69 (45%)	77 (55%)	.081
Diabetes	42 (27%)	40 (28%)	.804
Asthma	17 (11%)	13 (9%)	.62
Coronary artery disease	17 (11%)	33 (23%)	.004**
Chronic obstructive pulmonary disease	10 (6%)	19 (13%)	.042*
Congestive heart failure	8 (5.19%)	17 (12.14%)	.033*
Cancer	7 (4.55%)	11 (7.86%)	.237
Immunosuppression	13 (8.44%)	12 (8.57%)	.968
Chronic kidney disease	14 (9.09%)	19 (13.57%)	.224
**Lab values at admission**			
Alanine aminotransferase	36 (24, 60)	37 (21, 58)	.891
Vitamin D1	22.0 (21.9, 22.0)	22 (22.0, 22.0)	.082
Aspartate aminotransferase	45 (30, 72)	55 (32, 87)	.139
Bicarbonate	23 (21, 25)	22 (20, 25)	.086
Brain natriuretic peptide	155 (52, 705)	712 (227, 2826)	.0001***
C-reactive protein	10 (5, 18)	13 (6, 22)	.112
Creatinine	1.0 (0.8, 1.5)	1.3 (0.9, 2.5)	.008**
D-dimer	337 (225, 510)	506 (316, 1424)	.0005***
Ferritin	899 (400, 1865)	986 (392, 1696)	.628
Hematocrit	42 (36, 46)	41 (36, 44)	.499
Lactate dehydrogenase	398 (290, 537)	462 (346, 649)	.017*
Lymphocytes	11 (8, 16)	9 (6, 16)	.06
Sodium	135 (132, 139)	137 (133, 139)	.179
Procalcitonin	0.2 (0.1, 0.6)	0.4 (0.2, 1.0)	.01**
Troponin	0.01 (0.01, 0.01)	0.01 (0.01, 0.05)	.015*
White blood cells	7 (6, 10)	9 (6, 13)	.054
**Vital signs at admission**			
Systolic blood pressure	125 (115, 140)	128 (116, 146)	.327
Diastolic blood pressure	73 (67, 79)	71 (65, 77)	.155
Heart rate	91 (82, 105)	93 (83, 106)	.83
Body temperature	37.5 (37.1, 38.0)	37.3 (36.9, 37.8)	.016*
Oxygen saturation (SpO_2_)	95 (93, 97)	94 (92, 96)	.047*
Respiratory rate	22 (18, 24)	23 (19, 28)	.029
**Arterial blood gas at admission**			
HCO_3_	23 (21, 26)	22 (20, 26)	.819
PaCO_2_	42 (37, 50)	49 (37, 67)	.212
PaO_2_	123 (90, 184)	94 (81, 145)	.379
pH	7.36 (7.26, 7.42)	7.30 (7.21, 7.37)	.043*

Note: Group comparison of categorical variables in frequencies and percentages used χ^2^ test or Fisher exact tests. Group comparison of continuous variables in medians and interquartile ranges used the Mann–Whitney *U*-test. Values in percentages for age and laboratory data represent 95% CI and are simple proportions for all others.

Abbreviations: COVID-19, coronavirus disease of 2019; MV, mechanical ventilation; PaO_2_, measured the partial pressure of oxygen in arterial blood; PaCO_2_, measured the partial pressure of carbon dioxide in arterial blood; HCO_3_, calculated concentration of bicarbonate in arterial blood.

*P < .05, **P < .01, ***P < .001.

**Figure 2.**
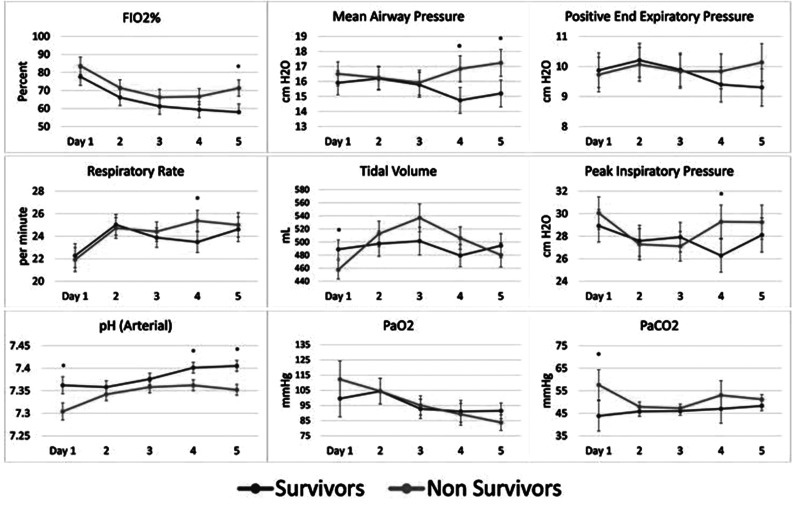
First 5 days MV parameters and ABG values for survivors and nonsurvivors. Error bars are SEM. *P < .05 (repeated ANOVA).

Abbreviations: MV, mechanical ventilation; ABG, arterial blood gas; SEM, standard error of the mean; ANOVA, analysis of variance

Figure 2 plots the MV parameters for survivors and nonsurvivors over the first 5 days. By the end of this timeframe, nonsurvivors were receiving significantly higher levels of FIO_2_% (day 5, [*P* < .05]), reached higher levels of MAP (days 4 and 5), peak inspiratory pressure (day 4), and respiratory rate (day 4) compared to survivors (*P* < .05). Nonsurvivor pH values were lower, showing statistical significance at days 1, 4, and 5 (*P* < .05). PaO_2_ did not differ significantly between groups. PaCO_2_ was significantly higher only on day 1 in the nonsurvivors (*P* < .05), though not in subsequent days, likely reflecting efforts by their physicians to regulate gas exchange.

**Figure 3.**
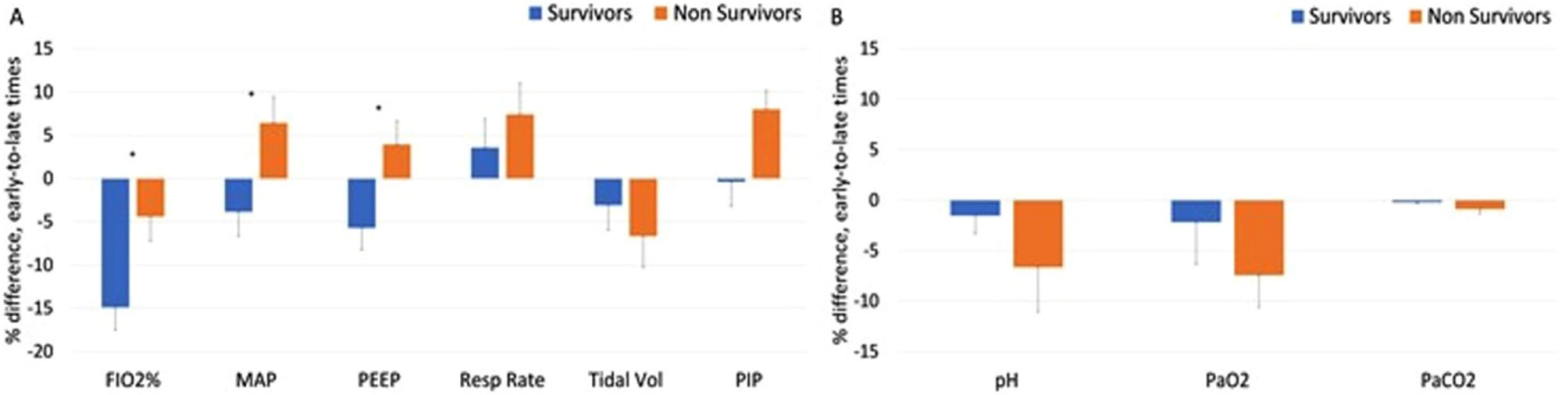
Differences between early time points (mean of days 1-3) and late time points (mean of days 4-5) on MV, for survivors and nonsurvivors. (A) Ventilation parameters and (B) ABG values. Error bars are SEM. *P < .05 (MAP = mean airway pressure, PEEP = positive end expiratory pressure, PIP = peak inspiratory pressure).

Abbreviations: MV, mechanical ventilation; ABG, arterial blood gas; SEM, standard error of the mean.

Based on these observations, we grouped the data into early (days 1-3) and late (days 4-5) time points and expressed them as percent changes between early and late time points (“temporal changes”). The temporal changes in PEEP and MAP differed significantly between nonsurvivors and survivors, showing an increase in nonsurvivors and a decline in survivors (*P* < .05). The temporal change in FiO_2_% of nonsurvivors was significantly less negative than those of survivors (*P* > .05). The temporal changes in RR, TV, and PIP were not significantly different between the groups, though all trended toward more intense ventilation requirements in the nonsurvivor group compared to survivors (Figure 3A). There were also no group temporal changes for PaO_2_, PaCO_2_, and pH (Figure 3B).

**Figure 4.**
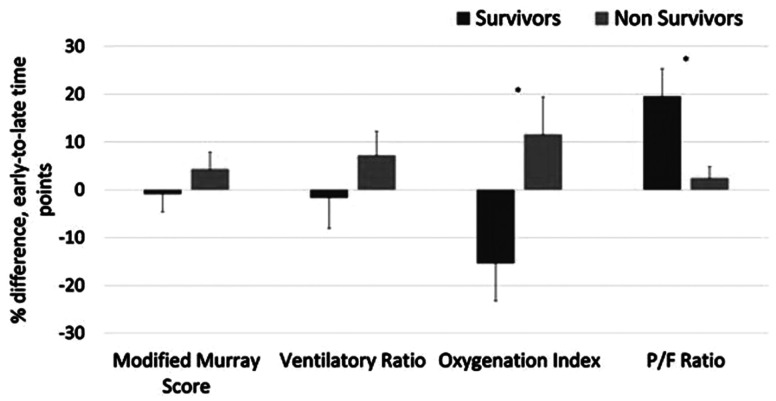
The differences between early and late time points for Murray score, ventilatory ratio, oxygenation index, and P/F ratio for COVID-19 survivors and nonsurvivors. All error bars are based on SEM. *P < 0.05.

Abbreviations: P/F, PaO_2_/FiO_2_; COVID-19, coronavirus disease of 2019; SEM, standard error of the mean.

One hundred eighty patients (61%) had every variable needed to calculate OI, P/F, MLIS, and VR for early and late time points and were included in Figure 4. Figure 4 illustrates that nonsurvivors developed substantially worsening OI and P/F ratios over the 5 days of MV (*P* < .05). The magnitude of temporal change in MLIS and VR did not differ significantly between survivors and nonsurvivors during the 5 days.

We evaluated the mortality standardized OR of several clinical variables (Table 2), inclusive of 208 patients. ORs were adjusted for the demographic variables and co-morbidities that showed the greatest individual differences between groups: age, male gender, HTN, and CAD. The adjusted OR for mortality was statistically significant for 3 variables: age, OR = 1.82 (95% CI 1.28-2.59, *P* = .001); OI at days 4 to 5, OR = 2.26 (95% CI 1.35-3.78, *P* = .002); and OI percent change, OR = 1.90 (95% CI 1.12-3.22, *P* = .02). The adjusted OR for mortality approached significance for OI at days 1 to 3, OR = 1.57 (95% CI 0.97-2.52, *P* = .06), and P/F ratio at days 4 to 5, OR = 0.66 (95% CI 0.42-1.43, *P* = .07). We did not detect collinearity between variables and did not find interactions between clinical variables or between clinical variables and comorbidities.

**Table 2.** Adjusted Mortality Odds Ratio for Demographic Variables and for the Mean Values of Calculated Early Scores (Days 1-3), Late Scores (Days 4-5), and Percent Change Over Time (Early to Late).

	Adjusted odds ratio (95% CI)	Adjusted *P* value
**Murray lung injury score**		
Days 1-3	1.10 (0.74-1.63)	.64
Days 4-5	1.11 (0.69-1.77)	.68
% change, early to late	1.06 (0.66-1.70)	.82
**Ventilatory ratio**		
Days 1-3	1.31 (0.83-2.09)	.25
Days 4-5	1.01 (0.68-1.50)	.97
% change, early to late	0.85 (0.76- 4.12)	.38
**Oxygenation index**		
Days 1-3	1.57 (0.97-2.52)	.06
Days 4-5	2.26 (1.35-3.78)	.002**
% change, early to late	1.90 (1.12-3.22)	.02*
**P/F ratio**		
Days 1-3	0.79 (0.49-1.28)	.34
Days 4-5	0.66 (0.42-1.03)	.07
% change, early to late	0.72 (0.47-1.10)	.13
Age	1.82 (1.28-2.59)	.001**
Hypertension	0.97 (0.52-1.82)	.92
Coronary artery disease	1.88 (0.78-4.57)	.16
Male gender	1.71 (0.90-3.26)	.10
Chronic obstructive pulmonary disease	1.99 (0.63-6.29)	.24
Congestive heart failure	0.78 (0.23-2.65)	.69
Diabetes	1.05 (0.55-2.02)	.87
Asthma	0.96 (0.40-2.33)	.94
Cancer	0.63 (0.17-2.35)	.50
Chronic kidney disease	0.70 (0.24-2.04)	.51
Immunosuppression	0.77 (0.22-2.70)	.68

Note: Adjusted confidence intervals account for the top demographic variables as potential confounders: age, gender, prior hypertension, and prior coronary disease.

*P < .05, **P < .01.

### Ventilator-Free Days

We computed the Spearman rank-order correlation of calculated scores with VFDs (within the first 28 days after intubation). The number of VFDs correlated significantly with late modified Murray score (−0.22, *P* < .05), VR (−0.20, *P* < .05), early OI (−0.21, *P* < .01), late OI (−0.26, *P* < .01), early P/F ratio (0.21, *P* < .01), and late P/F ratio (0.28, *P* < .01) time points (Table 3).

**Table 3.** Correlation of Calculated Scores With Ventilator-Free Days at Early and Late Time Points.

Correlation with ventilator-free days	Early time point	Late time point
Modified Murray score	−0.054	−0.197
Ventilatory ratio	−0.046	−.166*
Oxygenation index	−.216**	−.278**
P/F ratio	.158*	.283**

*Definition of ventilator-free days:* 28 minus the number of days on mechanical ventilation in the first 28 days, starting at the date of intubation. If a patient died within that time or remained on ventilation past 28 days they received a value of 0 for this variable. *P < .05, **P < .01.

## Discussion

In this cohort, COVID-19 patients with respiratory failure, we explored the relationship between mortality and gas exchange during the first 5 days of MV. The major findings are:

- After day 3 of MV, the FiO_2_ and airway pressures used in nonsurvivors began to increase relative to survivors, though the trend in PaO_2_ did not differ between groups. That is, compared to survivors, nonsurvivors required a substantially greater intensity of MV over time to sustain the same level of oxygenation. This is reflected in the temporal trends in OI, which significantly improved in survivors and significantly worsened in nonsurvivors at the later time points (days 4 and 5 of MV). The P/F ratio also improved more in survivors than in nonsurvivors; however, its discriminatory value was lower, since it improved in both survivors and nonsurvivors and the magnitude of the difference was small (15 points).
  The OI represents the “pressure cost” of oxygenation and is conceptually a more robust index of gas exchange than the P/F ratio.^7,16^ The latter can be artifactually changed by raising or lowering mean airway pressure (by changing PEEP for example) to recruit or de-recruit diseased lung units. The OI takes into account airway pressure and thereby reflects both the magnitude of O_2_ exchange and the intensity of ventilator support required to achieve it. It varies inversely with the P/F ratio; a higher OI value indicates worse gas exchange. Guidelines published by the Pediatric Acute Lung Injury Consensus Conference Group in 2015 strongly recommend using the OI rather than the P/F ratio as the primary metric of severity in pediatric ARDS.^17^ In pediatric literature on ARDS, a markedly elevated OI despite optimal MV is widely accepted as a poor prognostic marker and an indication to begin extracorporeal membrane oxygenation.^18,19^ However, because of the extensive use of the P/F ratio in adult research in ARDS, many physicians have not transitioned to using this valuable and nuanced marker of gas exchange. OI is readily derived from data that is routinely charted in most electronic medical records.
- We observed that an elevated OI at the later time points was strongly associated with mortality (OR 2.26) in the multivariable model, and correlated with fewer VFDs, at both the early and late time points. Notably, the OI did not change significantly between days 4 and 5, and thus served as a persisting marker of deteriorating gas exchange among nonsurvivors. We did not find that the P/F ratio was statistically correlated to mortality.
  Prior reports of ARDS in non-COVID patients have shown that OIs such as OI and P/F ratio are variably and only weakly associated with mortality, with ORs ranging from 1.0 to 1.8, while clinical factors such as age, organ failure scores, and active malignancy are more strongly predictive of mortality.^16,20,21^ This supports the premise that death from non-COVID-related ARDS is closely related to nonpulmonary organ failure, and not closely related to gas exchange failure per se.^8,16,22,23^ In contrast, a recent cohort study of COVID-19 patients in Michigan found that pulmonary dysfunction itself was the primary cause of death in 56% of COVID-19 patients, compared to 22% of those with respiratory failure of other causes.^24^ Along these same lines, a multivariate analysis of ventilated COVID-19 patients in the Netherlands found that lower PaO_2_/FiO_2_ was associated with fewer VFDs by day 28.^25^ Our results confirm and extend these findings, showing that in COVID-related respiratory failure, worsening OI after 3 days of MV is itself strongly and independently associated with higher mortality and fewer VFDs.
- Hypercapnia (high PaCO_2_) was substantially worse on day 1 of MV in nonsurvivors compared to survivors, even though minute ventilation differed by only 8% at that time point. The product of minute ventilation and PaCO_2_ is the basis of the VR; it reflects the intensity of MV required to excrete CO_2_ in the lungs. A high VR represents either inefficient ventilation due to dead space, or high metabolic production of CO_2_. In our exploratory analysis, we compared VR between survivors and nonsurvivors on day 1 of MV. The VR was worse among our nonsurvivors, though this did not reach statistical significance (OR 2.34, *P* = .17). Thus, we did not demonstrate clear evidence of inefficient CO_2_ excretion among nonsurvivors compared to survivors. Prior investigators have found that VR and calculation of dead space by the Bohr-Enghoff equation are each independently associated with mortality in patients with early ARDS, with ORs ranging from 1.20 to 1.45.^26,27^ An elevated dead space may occur in COVID-19 due to occlusion of lung capillaries by in-situ thrombosis resulting from a viral coagulopathy that is specific to SARS-CoV-2.^28^ Pathologic evidence of this process is seen in autopsy studies of patients dying of COVID-19, showing a high incidence of both lung capillary microthrombi and large vessel pulmonary emboli.^29,30^ The initial hypercarbic acidosis among our nonsurvivors may simply be a sign that the onset of MV was inappropriately delayed. Alternatively, it may indicate that the metabolic production of CO_2_ was greater in nonsurvivors. The data collected in our study does not allow us to distinguish these potential causes of excess early hypercapnia in nonsurvivors.
- The MLIS showed no significant difference between early and late time points for survivors and nonsurvivors. There was no significant association between MLIS and mortality at either the early or later time points.

Our observations suggest that day 4 of MV represents an inflection point in gas exchange among ventilated COVID-19 patients. A worsening OI at this point may provide an early warning sign of poor outcomes. We stress that these retrospectively derived findings are exploratory in nature. Further work is required to determine if the temporal change in OI can be incorporated in a predictive model of outcomes in severe COVID-19 patients.

## Limitations

Our study had several limitations. First, this was a retrospective cohort study and as such potentially confounding factors may have been overlooked. To avoid overfitting the model, only a limited number of clinical variables were entered into the logistic regression; it is possible that potentially relevant variables were not evaluated. We included 4 variables known to significantly impact mortality in COVID-19: age, male gender, and the presence of hypertension or coronary disease. These were the 4 variables that in our data showed the greatest individual ORs. Second, a study of this size may have had insufficient power to detect real differences in the association of outcomes with VR or other parameters. Third, data were obtained from a single hospital's COVID-19 database. We cannot be certain our results will generalize to other hospital settings.

## Conclusion

We evaluated 4 commonly used scores of gas exchange and lung injury in COVID-19 patients with ARDS over the first 5 days of MV. Nonsurvivors of COVID-19 needed increasing airway pressures and FiO_2_ to sustain gas exchange. This is reflected in the OI which worsened in nonsurvivors and improved in survivors over this timeframe. The temporal change in OI was the variable that most clearly differentiated survivors from nonsurvivors. A worsening OI after day 3 of MV may have value as a marker of poor outcome in respiratory failure due to COVID-19.
